# Fully automated radiosynthesis of [^18^F]LBT999 on TRACERlab FX_FN_ and AllinOne modules, a PET radiopharmaceutical for imaging the dopamine transporter in human brain

**DOI:** 10.1186/s41181-020-00105-w

**Published:** 2020-11-16

**Authors:** Christine Vala, Céline Mothes, Gabrielle Chicheri, Pauline Magadur, Gilles Viot, Jean-Bernard Deloye, Serge Maia, Yann Bouvet, Anne-Claire Dupont, Nicolas Arlicot, Denis Guilloteau, Patrick Emond, Johnny Vercouillie

**Affiliations:** 1 Zionexa, 75017 Paris, France; 2 Cyclopharma, 63360 Saint-Beauzire, France; 3 CERRP, 37100 Tours, France; 4 UMR 1253, iBrain, Université de Tours, Inserm, 37000 Tours, France; 5 INSERM CIC 1415, University Hospital, 37000 Tours, France; 6 CHRU de Tours, services de Médecine Nucléaire in vitro et in vivo, 37000 Tours, France

**Keywords:** Automation, Radiosynthesis, [^18^F]LBT999, PET, Dopamine transporter, Parkinson’s disease

## Abstract

**Background:**

Fluorine labelled 8-((*E*)-4-fluoro-but-2-enyl)-3β-*p*-tolyl-8-aza-bicyclo[3.2.1]octane-2β-carboxylic acid methyl ester ([^18^F]LBT999) is a selective radioligand for the in vivo neuroimaging and quantification of the dopamine transporter by Positron Emission Tomography (PET). [^18^F]LBT999 was produced on a TRACERlab FXFN for the Phase I study but for Phase III and a potent industrial production transfer, production was also implemented on an AllinOne (AIO) system requiring a single use cassette. Both production methods are reported herein.

**Results:**

Automation of [^18^F]LBT999 radiosynthesis on FXFN was carried out in 35% yield (decay-corrected) in 65 min (*n* = 16), with a radiochemical purity higher than 99% and a molar activity of 158 GBq/μmol at the end of synthesis. The transfer to the AIO platform followed by optimizations allowed the production of [^18^F]LBT999 in 32.7% yield (decay-corrected) within 48 min (*n* = 5), with a radiochemical purity better than 98% and a molar activity above 154 GBq/μmol on average at the end of synthesis. Quality controls of both methods met the specification for clinical application.

**Conclusion:**

Both modules allow efficient and reproducible radiosynthesis of [^18^F]LBT999 with good radiochemical yields and a reasonable synthesis time. The developments made on AIO, such as its ability to meet pharmaceutical criteria and to more easily comply with GMP requirements, make it an optimal approach for the potent industrial production of [^18^F]LBT999 and future wider use.

## Background

The dopamine transporter (DAT) is known to be involved in a number of physiological and pathological processes in the brain and has been extensively explored by molecular imaging techniques including single-photon emission computed tomography (SPECT) or positron emission tomography (PET). The exploration of DAT by SPECT or PET has demonstrated its interest in many diseases such as Parkinson’s disease, Huntington’s disease, depression, schizophrenia, attention deficit and hyperactivity disorders, or addictions (Arakawa et al., 2009; Brooks, 2016; da Silva et al., 2011; Hwang and Yao, 2013; Karila et al., 2016; Makinen et al., 2017; Varrone and Halldin, 2010, 2012; Zoons et al., 2017).

Currently, [^123^I]FP-CIT (or [^123^I]Ioflupane or DATSCAN™ from GE Healthcare) is commercially available and is used daily in nuclear medicine departments. [^123^I]FP-CIT is used for differential diagnosis between an essential tremor and an extra-pyramidal syndrome and to differentiate probable Lewy Bodies Dementia (LBD) from other dementia such as Alzheimer disease (Brigo et al., 2015; Darcourt et al., 2010). Despite its common use, an [^123^I]FP-CIT scan presents some disadvantages which can be restrictive for patients with movement disorders or dementia, such the duration of the examination (half a day) or the thyroid protection due to substantial release of Iodine-123. These drawbacks, associated with the desire to obtain better quality images, encouraged several groups to develop new radiotracers with a focus on PET technology.

**Fig. 1:**
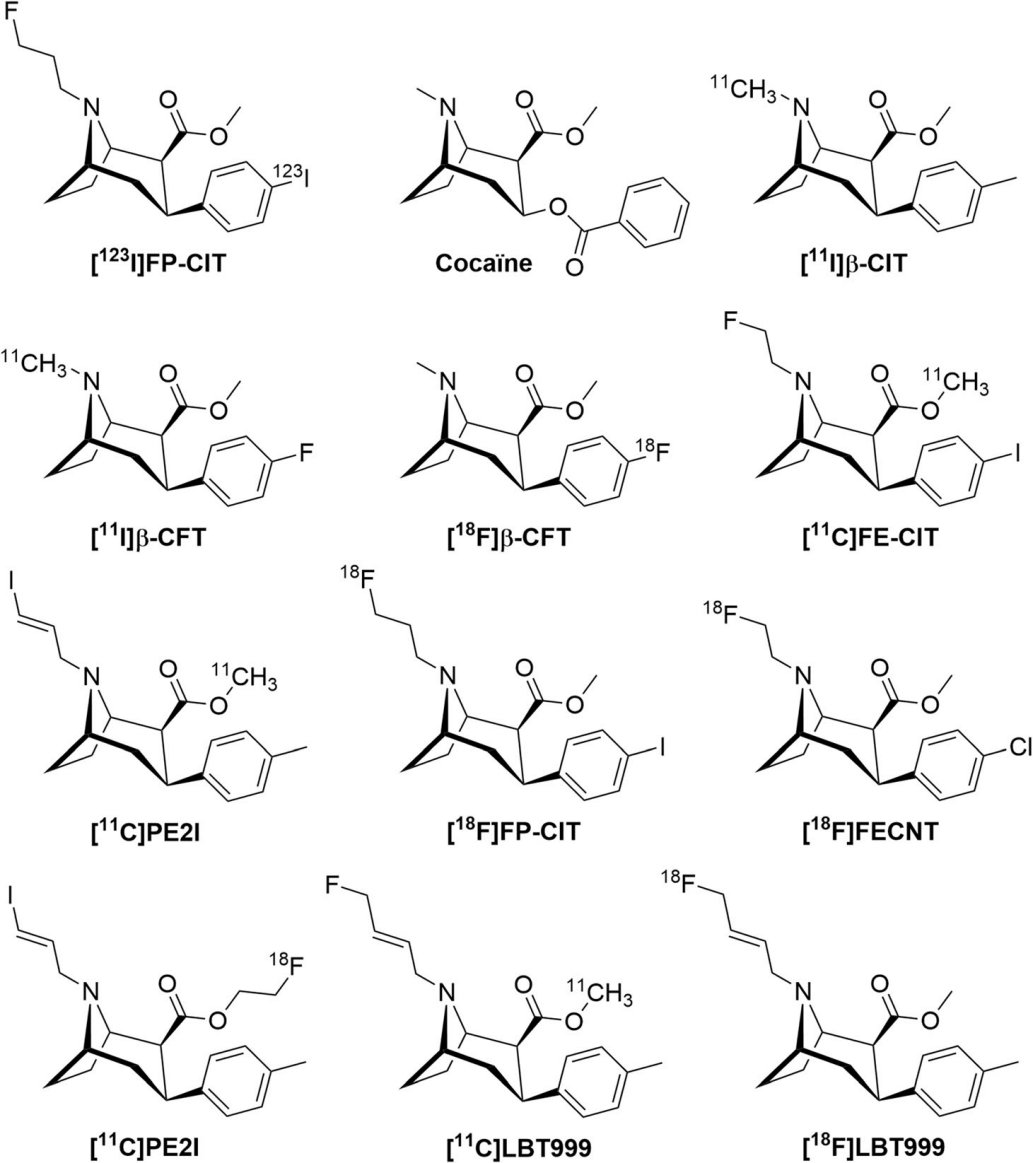
Chemical structures of various SPECT and PET radioligands for DAT imaging

The advantage of PET imaging over SPECT is its better resolution and sensitivity, affording a better quality of images and quantification analysis. For these reasons, many efforts have been made to develop a PET tracer labelled with Carbon-11 as a research tool or with Fluorine-18 in the hope of routine use. Among the developed radiotracers, cocaine and its derivatives, [^11^C/^18^F]β-CFT (Laakso et al., 1998; Wong et al., 1993), [^11^C]β-CIT (Farde et al., 1994), [^11^C]FE-CIT (Antonini et al., 2001), [^11^C]PE2I (Ribeiro et al., 2007),[^18^F]FP-CIT (Kazumata et al., 1998), [^18^F]FECNT (Davis et al., 2003), [^11^C/^18^F]FE-PE2I (Sasaki et al., 2012; Varrone et al., 2009) and [^11^C/^18^F]LBT999 (Dolle et al., 2006a; Saba et al., 2007; Serriere et al., 2014; Varrone et al., 2011) were synthesized, fully characterized or even evaluated within clinical trials. (Fig. 1).

Our contribution in this field was the development of [^18^F]LBT999 which displays in vitro a high affinity (K_D_ = 9 nM) and selectivity for DAT (over the norepinephrine and serotonin transporter (IC_50_ > 1000 nM)) (Chalon et al., 2006).

PET investigations of [^18^F]LBT999 in nonhuman primates showed a good brain uptake and biodistribution, which was consistent with the known region of DAT density (Varrone et al., 2011). Moreover, [^18^F]LBT999 showed an appropriate in vivo kinetic in rodent and nonhuman primates allowing DAT quantification in the striatum, the midbrain and thalamus (Serriere et al., 2014; Varrone et al., 2011).

Based on these encouraging results, a first-in-man study (NCT02393027) using [^18^F]LBT999 as a DAT agent was conducted in France. The objective of the study was to demonstrate the ability of [^18^F]LBT999 to easily discriminate healthy volunteers from parkinsonians by DAT imaging. The study revealed that the tracer allows a 10 min PET acquisition between 30 and 40 min post-injection (Arlicot et al., 2019; Ribeiro et al., 2020). In addition, no significant metabolites were observed at this time scan, confirming the potentiality of the tracer and thus motivating the set-up of a Phase III study.

**Fig. 2:**
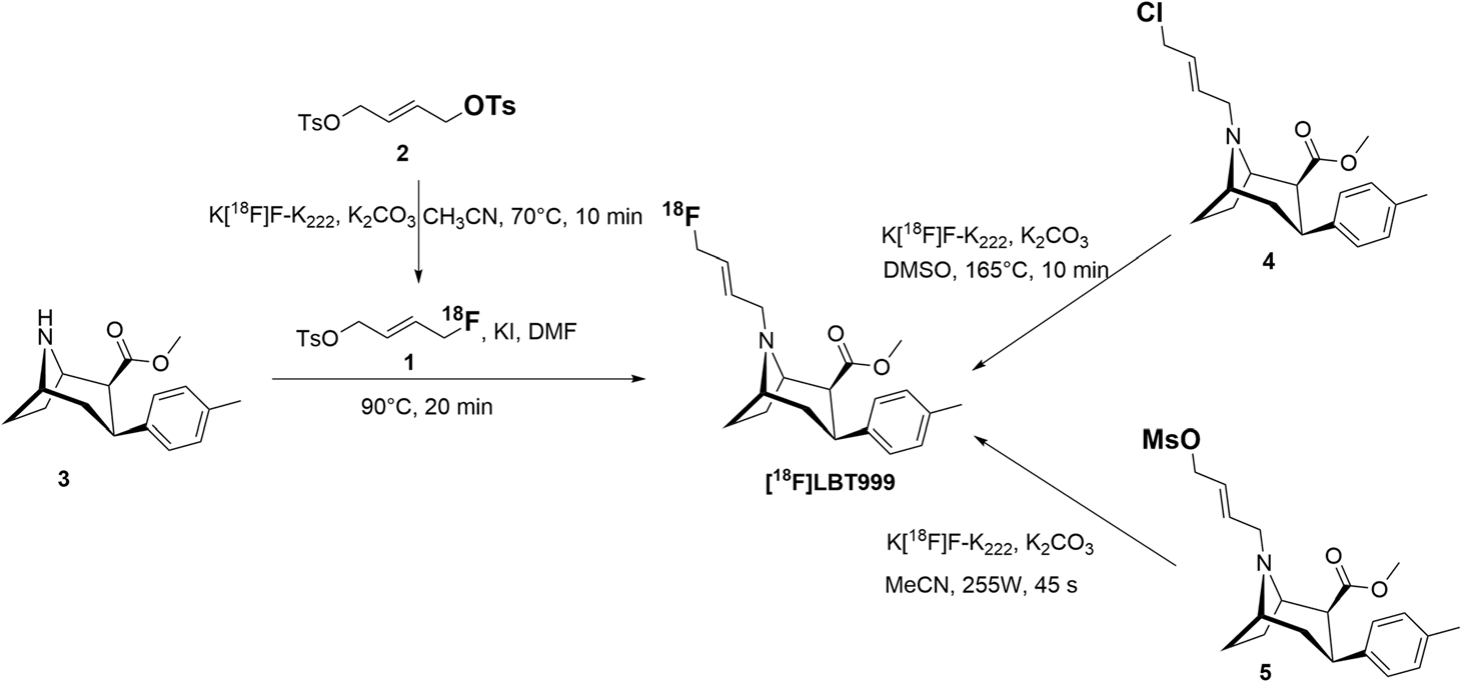
The two strategies of [^18^F]LBT999 radiosynthesis described in the literature (Dolle et al., 2008; Dolle et al., 2007; Riss and Roesch, 2009). A direct radiofluorination from chlorinated or mesylated precursor and a two-step approach

In the literature, two different strategies of radiosynthesis of [^18^F]LBT999 have already been reported (Fig. 2). The first one was a two-step radiochemical process (Dolle et al., 2006b), comprising the formation of (*E*)-1-fluoro-4-tosyloxybut-2-ene **1** from the ditosylate precursor **2**, followed by a *N*-alkylation reaction of the tropane derivative **3**. Typically, [^18^F]LBT999 was obtained in 95–100 min with an overall yield of 4.6–6.7% (non-decay corrected or 8.3–12.5% decay-corrected). The second one was a direct one-step radiosynthesis from a chlorinated precursor **4** (Dolle et al., 2008; Dolle et al., 2007). With these conditions, [^18^F]LBT999 was produced in 85–90 min with an overall yield of 10–16% (non-decay-corrected). In both cases, radiosynthesis of [^18^F]LBT999 was performed by using a computer-assisted Zymate-XP robot system (Zymark Corporation, USA) for radiosynthesis and purification, while the formulation of the final product was realized with a separate home-made Sep-pak Plus C18 device.

In order to improve the radiochemical yield of [^18^F]LBT999, a one-step strategy was also studied from a mesylate precursor **5** by using a microwave-assisted labelling method instead of conventional thermal heating (Fig. 2) (Riss and Roesch, 2009). In this case [^18^F]LBT999 was obtained in 27% non-decay corrected yield after labelling under constant microwave irradiation (255 W during 45 s). The yields were higher than previously reported but these conditions seem difficult to apply in clinical or industrial routine production as classical commercial modules are not equipped with a microwave and only a few R&D radiochemistry laboratories around the world use this method.

Based on Dollé et al.’s publications (Dolle et al., 2008; Dolle et al., 2007) and our preclinical work (Serriere et al., 2014), [^18^F]LBT999 was produced during the Phase I study on a TRACERlab FXFN automate (Arlicot et al., 2019; Ribeiro et al., 2020). For the subsequent Phase III, however, we decided to work on AIO, a single-use cassette module, to increase the batch size and to more easily comply with GMP standards. Compared to TRACERlab FXFN, the use of a cassette module affords several advantages such as securing production by the use of chemical kits, ensuring no cross-contamination with a single cassette use and not requiring any cleaning and validation of the cleaning procedure of the automate. Moreover, cassette modules are adapted to facilitate industrial transfer and to perform the industrial production of radiopharmaceuticals.

We report here the first fully automated radiosynthesis of [^18^F]LBT999 on TRACERlab FXFN and AIO modules in a one-step procedure starting from the corresponding chlorinated precursor. The full quality control validated for human injection is also described for both approaches.

## Materials and methods

All solvents, chemicals and reagents were purchased from commercial suppliers, namely Sigma Aldrich, Merck, ABX or VWR, and were used as received without further purification. Sterile water and saline (0.9%) for injection were respectively purchased from B. Braun and Aguettant. Sep-Pak® cartridges were purchased from ABX for pre-conditioned Light QMA Cartridge with potassium carbonate or from Waters Corporation for Accell Plus QMA Plus Light Cartridges (pre-conditioned with 10 mL of 0.5 M K_2_CO_3_ solution, followed by 20 mL of water) and *t*C_18_ Plus Short Cartridges (treated with 1 mL Ethanol and rinsed with 10 ml of water). Sodium Ascorbate (Na-Asc) was purchased from Cooper.

The cold [^19^F]LBT999 reference compound and its chlorinated precursor **4** (Fig. 2) were synthesized and supplied by Orphachem (Clermont-Ferrand, France), according to a procedure described in the literature (Dolle et al., 2008; Dolle et al., 2006a). The precursor and reference have been qualified as starting material or reference compound for radiopharmaceutical production. [^18^F]fluoride was produced by Cyclopharma Laboratories via the ^18^O(p,n)^18^F nuclear reaction with a GE cyclotron (PETtrace 800, 16.5 MeV). ^18^O-enriched water was purchased from CIL or Rotem. The bombardment of ^18^O-enriched water with protons at 80 μA during 30 min provided about 80 GBq of [^18^F]fluoride in ^18^O-enriched water (4 mL), then the activity was directly transferred under helium pressure to the radiosynthesis module.

Automated radiosynthesis of [^18^F]LBT999 was performed either on a TRACERlab FXFN module (GE) or on an AllinOne module (Trasis), including HPLC purification and formulation. Semi-preparative HPLC purification was carried out on an Alltima C18 column (250 X 10 mm, 5 μm, Grace, Alltech, France) for TRACER lab FX_FN_ and for the AIO on an Zorbax EclipseXDB-C18 (250 X 9.4 mm, 5 μm, CA, Agilent, USA). In both cases, ammonium acetate 0.1 M/acetonitrile: 40/60 was used as mobile phase at a 4 mL/min flow rate. Sodium ascorbate (0.5%) was added for purification after AIO process optimization.

For the formulation, the collected fraction was diluted with water, trapped on a SPE cartridge and rinsed with water. [^18^F]LBT999 was eluted from the cartridge by injectable ethanol and the formulation complete by adding saline (0.9%). The formulation was done to not exceed 10% of ethanol (v/v). Sodium ascorbate (0.5%) was added to the aqueous solution for the AIO process optimization. The final product was aseptically filtered (0.22 μm PES vented) into sterile vials for human injection. The filters were procured from RoweMed (A-6606) and sterile vials (12 mL) from Hospira.

For quality control, analytical HPLC analyses were performed by using two HPLC conditions with a gamma detection and UV signal detection at 220 nm. *HPLC a:* Equipment: ICS 3000 (Thermo Fisher Scientific); Column: μBondapak™ (C18, 3.9 X 300 mm, 10 μm); mobile phase: acetonitrile/water/TFA: 50/50/0.1 (v/v/v); Flow rate: 1 mL/min; *HPLC b*: Equipment: Ultimate 3000 (Thermo Fisher Scientific); Column: Brownlee CHOICE C18 (250 X 4.6 mm, 5 μm); mobile phase: ammonium acetate 0.1 M/acetonitrile: 35/65 (v/v); Flow rate: 1 mL/min.

Thin layer chromatography (TLC) analyses were carried out on silica gel 60 F_254_ (Merck) by using a radioTLC reader (BioscanB-MS-1000) and ethyl acetate as mobile phase.

Na-Asc concentration was determined by using a reflectometer (Reflectoquant®, RQflex® 10, EMD Millipore).

### Automated radiosynthesis of [^18^F]LBT999

For both modules, automated in-house programs were developed to synthesize the [^18^F]LBT999, including exactly the same sequence, fully described in Table 1, with four main steps: 1) Azeotropic drying of [^18^F]Fluoride, 2) [^18^F]radiofluorination of the precursor **4**, 3) HPLC purification and 4) Formulation.

**Table 1:** Common steps for the automated radiosynthesis of [^18^F]LBT999 on TRACERlab FXFN and AIO modules

**1) Azeotropic drying and complex [**^**18**^**F]KF/K222 formation**	1- [^18^F]Fluoride trapping on a QMA after production and transfer from the cyclotron
	2- Elution of [^18^F]Fluoride in the reactor with a solution of K_222_/K_2_CO_3_
	3- Azeotropic drying of [^18^F]Fluoride with successive addition of acetonitrile
**2) [**^**18**^**F]radiofluorination of 4**	1- Addition of the precursor **4**
	2- [^18^F]Radiofluorination
**3) Purification of [**^**18**^**F]LBT999**	1- Cooling and Dilution of the crude mixture with water or a solution of water in presence of Na-Asc (0.5%).
	2- Pre-purification on C18 and washing with water or a solution of water in presence of Na-Asc (0.5%).
	3- Elution of crude product and injection into the HPLC loop
	4- HPLC Purification
	5- Collection of the product
	6- Trapping on C18 cartridge and washing
**4) Formulation and dispensing of [**^**18**^**F]LBT999**	1- Elution with 10% EtOH and saline (0.9%) or saline (0.9%) with Na-Asc (0.5%)
	2- Transfer of formulated [^18^F]LBT999 and vial dispensing after sterile filtration

### TRACERlab FXFN module

The module was used in its basic configuration without any modifications. After the end of bombardment (EOB), the [^18^F]fluoride produced by the cyclotron was delivered to the already conditioned automate. Then, [^18^F]Fluoride was trapped on a Sep-Pak Accell Plus QMA Plus Light Cartridge to remove [^18^O]H_2_O. [^18^F]Fluoride was eluted into the reaction vessel using 0.9 mL of an aqueous solution of Kryptofix (K_2.2.2._, 7.2 mg) in 715 μL of acetonitrile and potassium carbonate (K_2_CO_3_, 3.8 mg) in 285 μL of water. The [^18^F]fluoride was dried by azeotropic distillation under vacuum and helium flow by heating at 70 °C to 100 °C. Following the drying step of [^18^F]fluoride, the chlorinated precursor **4** (3 mg) preliminary dissolved in 1 mL of anhydrous DMSO was added into the reaction vessel and heated at 165 °C for 10 min. After this time, the reactor was cooled to 50 °C and the crude reaction mixture was diluted with water (9 mL), passed through a Sep-Pak *t*C18 Plus Short Cartridge to remove unreacted [^18^F]fluoride and most polar impurities. The crude [^18^F]LBT999 was eluted from the cartridge by using acetonitrile (1 mL) prior to the semi-preparative HPLC purification. The [^18^F]LBT999 fraction was collected between 11 and 12 min, transferred into a flask preloaded with water (30 mL) then the resulting solution was passed through a Sep-Pak Alumina *N* Plus Light Cartridge and a Sep-Pak *t*C18 Plus Short Cartridge. The cartridges were rinsed with sterile water (5 mL) and [^18^F]LBT999 was eluted with injectable ethanol (1.5 mL) into the final product vial, containing 13.5 mL of saline (0.9%).

### Automated radiosynthesis of [^18^F]LBT999 on AIO

For the radiosynthesis of [^18^F]LBT999 on AIO, a single-use cassette was designed with an appropriate home-made program, based on the work previously developed on TRACERlab FXFN with some modifications. As usual with the AIO, before receiving the activity from the cyclotron, the cassette was placed on the platform and all reagents loaded on it.

At EOB, the [^18^F]fluoride was delivered and trapped on a preconditioned QMA carbonate cartridge, and eluted into the reaction vessel with 0.9 mL of a solution of Kryptofix (K_2.2.2._, 7.2 mg) in 715 μL of acetonitrile and K_2_CO_3_ (3.8 mg) in 285 μL of water. Then, the [^18^F]fluoride was dried by azeotropic distillation under vacuum and nitrogen flow by heating with successive addition of acetonitrile. The precursor **4** (6 mg) in DMSO (0.8 mL) was added to the dried residue (^18^F]fluoride/K_2.2.2._ complex) and after heating at 155 °C for 7.5 min, the reaction mixture was diluted in Na-Asc (0.5% in water, 9 mL). The resulting mixture was subsequently passed through a Sep-Pak *t*C18 cartridge, rinsed with water and the product was eluted with 1 mL of acetonitrile and mobile phase for injection into the HPLC loop for purification by semi-preparative HPLC. The [^18^F]LBT999 peak was detected between 11 and 12 min, collected and then diluted into 40 ml of Na-Asc (0.5% in water) and the resulting solution passed through a Sep-Pak *t*C18 cartridge and rinsed with water. The [^18^F]LBT999 was eluted with injectable EtOH (2.5 mL) and the formulation was completed with saline (0.9%) containing 0.5% of Na-Asc, affording 25 mL of mother solution.

### Quality control for human use

Visual inspection, testing for pH, filter integrity via the bubble test, radionuclide identity and purity tests via half-life and energy spectrum, radiochemical purity and identity by HPLC, chemical purity by HPLC, K_2.2.2._ spot test, sodium ascorbate concentration (reflectometer), bacterial endotoxin, the residual solvents analysis by GC, and sterility were performed following standard QC rules for fluorinated PET tracers.

### Stability of the formulation

The stability of [^18^F]LBT999 was verified by analytical HPLC and radioTLC at room temperature (20 °C) up to 8 h after the end of synthesis and on the day after production (data not reported).

## Results and discussion

In the present work, the first successfully automated radiosynthesis of [^18^F]LBT999 was reported on two commercial modules, GE TRACERlab FXFN and AIO up to the final dispensation, with the aim of conducting multicenter trials (Phase III) and industrial production in a later phase. Until now, [^18^F]LBT999 was exclusively synthetized and used in the framework of preclinical studies without any complete automated radiosynthesis reported in the literature.

### Comparison of AIO and GE TRACERlab FXFN production

**Fig. 3:**
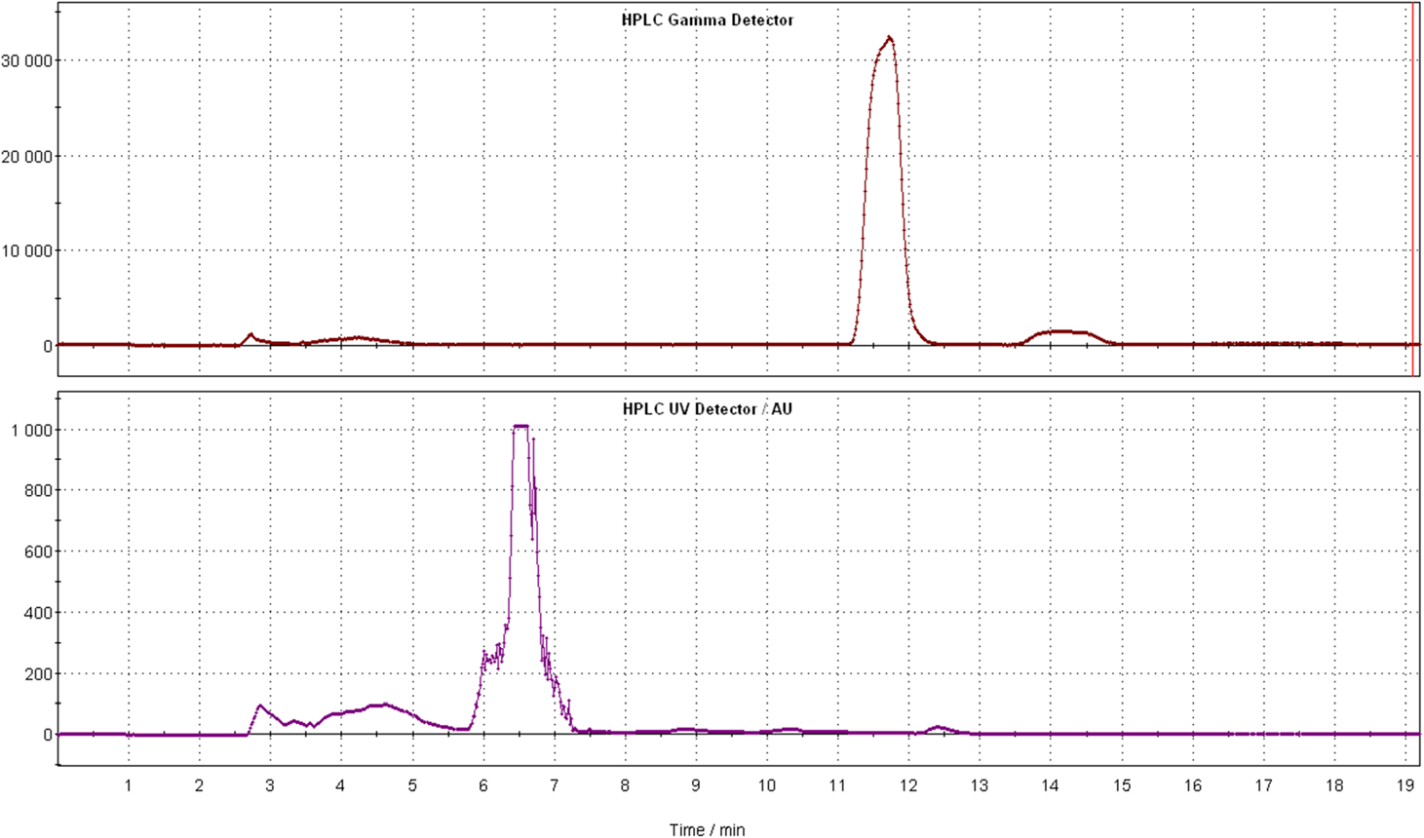
Chromatograms of semi-preparative HPLC [^18^F]LBT999 purification on TRACERlab FXFN modules (Gamma and UV). Alltima C18 column (250 X 10 mm, 5 μm, Grace, Alltech, France) by using as mobile phase: ammonium acetate 0.1 M/acetonitrile: 40/60 at a 4 mL/min flow rate (RT = 11–12 min)

For the first-in-man investigations of [^18^F]LBT999 in France, automation was realized on a TRACERlab FXFN module based on the one-step radiofluorination from the chlorinated precursor **4**, by applying the conditions described in Serrière et al. (Serriere et al., 2014; Varrone et al., 2011) and Dollé et al. (Dolle et al., 2008; Dolle et al., 2007; Dolle et al., 2006b), with minor modifications. From these conditions, at the end of synthesis, the radiochemical yield of [^18^F]LBT999 was 35.3 ± 5.1% (23.3% activity yield) and the total synthesis duration was 65.8 ± 3.8 min (*n* = 16). Typically, starting from 77.2 GBq of [^18^F]Fluoride, 16.8 ± 1.9 GBq of [^18^F]LBT999 were produced, which is sufficient for clinical routine production. In comparison with the originally reported synthesis of Dollé et al. (Dolle et al., 2007), the full automation on the TRACERlab FXFN resulted in a shorter synthesis time, 65.8 min instead of 85–90 min, and an almost doubled radiochemical yield (23.3% decay-corrected compared to 10–16%, Table 2), even when starting from a higher activity amount (77.2 GBq instead of 37 GBq). HPLC conditions were modified compared to Dollé’s work (Dolle et al., 2007) to avoid TFA in the mobile phase. These conditions allowed an efficient purification and gave a similar profile (Fig. 3) to that reported by Dollé et al. (Dolle et al., 2007).

**Table 2:** Comparison of the main steps in the preparation of [^18^F]LBT999 on TRACERlab FXFN and AIO modules and synthetic results compared to the initial work by Dollé’s group (Dolle et al., 2007)

Synthetic results of production of [^18^F]LBT999	Dollé’s work (*n* > 20)	FXFN (*n* > 16)	AIO (*n* = 5)
***1. Azeotropic drying***			
*K*_*2.2.2*_*.*	12–15 mg	7.2 mg in 715 μL ACN	7.2 mg in 715 μL ACN
*K*_*2*_*CO*_*3*_	1 mL of a 1.0/mL solution	3.8 mg in 285 μL H2O	3.8 mg in 285 μL H2O
*Evaporation/Temperature*	145–150 °C 10 min	70–100 °C 10 min	95 °C 10 min
***2. Radiofluorination***			
Precursor	3.5–4.5 mg	3 mg	5.5–6 mg
DMSO	0.6 mL	1 mL	0.8 mL
Reaction time	10 min	10 min	7 min 30
Temperature	165 °C	165 °C	155 °C
***3. HPLC Purification***			
Eluent	H_2_O/CH_3_CN/TFA: 72/28/0.1	AcNH_4_ (0.1 M)/ CH_3_CN: 40/60	AcNH_4_ (0.1 M)/ CH_3_CN: 40/60 + Na-Asc (0.5%).
Flow rate	5 mL/min	4 mL/min	4 mL/min
*Column*	Symmetry®C18, Waters (300 × 7.8 mm, 7 μm)	Alltima® C18, Grace (250 X 10 mm, 5 μm)	Agilent Zorbax Eclipse XDB-C18 (250 X 9.4 mm, 5 μm)
***4. Formulation***			
ETOH	2.0 mL	1.5 mL	2.5 mL
NaCl (0.9%)	8.0 mL (volume adjusted bring EtOH concentration below 10%)	13.5 mL	22.5 mL + Na-Asc (0.5%).
**Synthetic results**			
Starting activity (GBq)	37	77.2 ± 2.1	102.7 ± 19
Radioactivity at EOS (GBq)	3.7–5.92	16.8 ± 1.9	23.8 ± 7.1
Radiochemical yield at EOS (%)	17.3–27.7	35.3 ± 5.1	32.7 ± 5.9
Activity yield at EOS (GBq)	10–16	23.3 ± 4.3	23.1 ± 4.5
Radiochemical purity (EOS)	> 95	99.4 ± 0.2	98.3 ± 0.5
Total synthesis time (min)	85–90	65.8 ± 3.8	48 ± 1.9
Molar activity at EOS(GBq/μmol)	37–111	158.6 ± 79.8	154.3 ± 51.1

For the Phase III project, the automation of [^18^F]LBT999 on the AIO system was implemented and optimization of the parameters (reaction time, T°, precursor amount, purification conditions…) was performed. Optimization of the parameters was required due to the different configuration of the synthesizers but also to a 32% increase in the starting activity (Table 2).

**Fig. 4:**
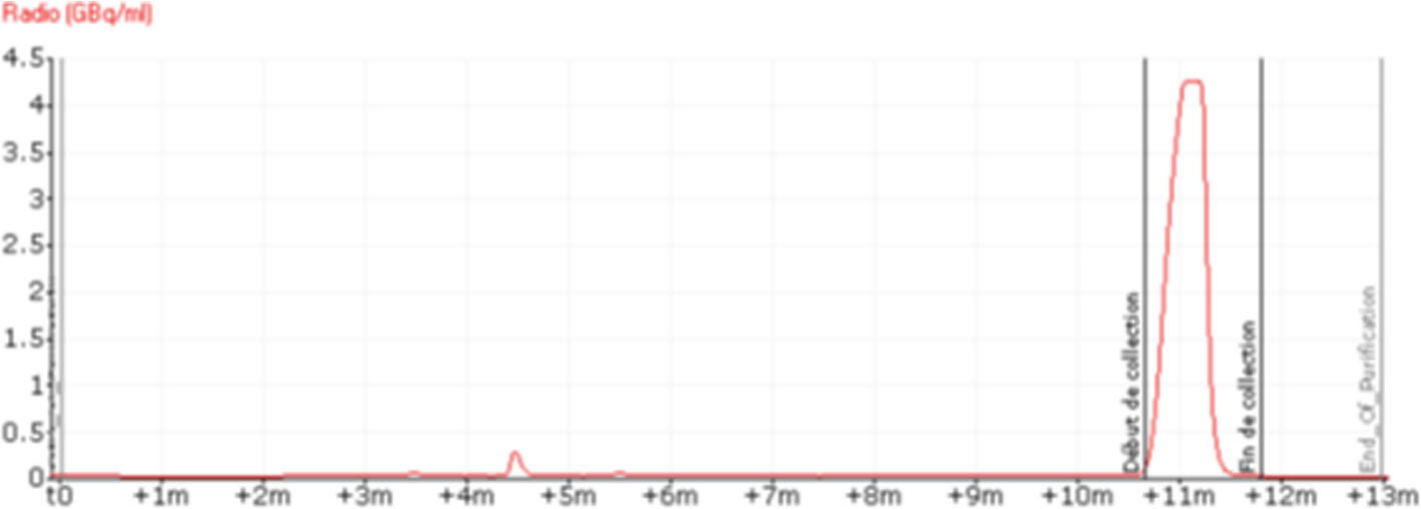
Semi-preparative HPLC radiochromatogram of [^18^F]LBT999 purification on AIO module: mobile phase and flow rate, ammonium acetate 0.1 M/acetonitrile: Agilent Zorbax EclipseXDB-C18 (9.4 × 250 mm, 5 μm, CA, USA), 40/60 at 4 mL/min in presence of Na-Asc (0.5%) (RT = 10.5–12)

Thus, regarding temperature, we found that 155 °C gave similar radiochemical yields to those observed at 165 °C with fewer side products formed. On the other hand, working at temperatures below 155 °C resulted in less fluorine-18 incorporation (data not shown). We also observed better radiochemical yields after 7.5 min than at 10 min reaction mainly due to a lower degradation of the precursor and [^18^F]LBT999. Moreover, using 6 mg of precursor instead of 3 mg contributed to increasing the incorporation yield, while a higher amount up to 10 mg did not significantly change the incorporation yield. Although doubling the amount of precursor and an increase in activity improved the incorporation of ^18^F on the LBT 999 precursor, we observed a lower purification efficiency with radiolabeled and non radiolabeled side products before the [^18^F]LBT999 peak. For these reasons we modified the HPLC conditions by replacing the Altima column by a Zorbax one (Fig. 4). This modification, combined with the addition of sodium ascorbate (0.5%) in the mobile phase and its addition as early as possible in the process after the dilution of the crude medium, allowed us to achieve a satisfactory purification efficiency, 98.3% instead of the 96% previously observed (data not reported). This purity can be maintained provided that radioactivity concentration is kept below 1.5 GBq/mL, otherwise the radiochemical purity drops, probably due to radiolysis. For this reason, the volume of mother solution on the AIO was increased to 25 mL instead of the 15 mL with the TRACERlab FXFN.

The combined improvements were used to produce 5 batches dedicated to the Phase III IMPD dossier (Table 2). These optimizations of the different parameters on the AIO system allowed us to prepare [^18^F]LBT999 in a total synthesis time of 48 ± 1.9 min with a 32.7 ± 5.9% radiochemical yield. Thus, starting from 102.7 GBq of [^18^F]Fluoride, 23.8 ± 7.1 GBq of [^18^F]LBT999 were produced (Table 2). While the RCY obtained with the AIO are similar or even a little bit lower than those observed on the GE FXFN, the automation on AIO resulted in a synthesis time that was 17 min shorter. Moreover, the production on AIO does not require the cleaning and validation of cleaning procedure as is required for the TRACERLAB FXFN. This results in a simplified installation/conditioning of the synthesizer and avoids any misfiling of vials that can be encountered on the TRACERLAB FXFN. The production of [^18^F]LBT999 on the AIO will more easily comply with a GMP production and will be better adapted to a potent future industrial production.

### Quality controls

Quality controls were performed on vials after dispensing for all the validated productions, tests or batches. QC revealed that the [^18^F]LBT999 met all acceptance criteria for clinical use (Table 3).

**Table 3:** Description and synthetic results of Quality control tests of [^18^F]LBT999 for human injection after radiosynthesis on TRACERlab FXFN and AIO modules

Quality control tests	Description of the test	Release criteria	QC Results of [^18^F]LBT999	
			FXFN	AIO
Particulates and color	Visual inspection for color & particulates	Clear, particle-free and colorless	passed	passed
pH	Use of pH strip test	4–5-8.5	5.8	7
Radionuclidic identity	Half-life determination	105–115 min	passed	passed
Radionuclidic purity	Gamma spectrometer	505-515 keV	passed	passed
Filter integrity	Bubble point test	Meet manufacturer’s requirement	passed	passed
Radiochemical purity	Determined by HPLC	> = 95%	> 99.2	> 98
Radiochemical identity	Determined by HPLC	+/− 3% rt	passed	passed
Radiochemical identity	Determined by Radio-TLC	> = 95%	passed	passed
Residual Kryptofix 2.2.2	Color spot test	< 0.22 mg/mL	passed	passed
Na-Asc concentration	Reflectometer	0.35–0.47% m/V	passed	passed
Residual solvent: ACN	Determined by GC	<= 0.41 mg/ml	passed	passed
Residual solvent: EtOH	Determined by GC	<= 100 mg/ml	passed	passed
Bacterial endotoxin	Endosafe (Charles River)	<= 17.5 EU/dose	passed	passed
Sterility test*	No growth observed after 14 days	No visible μorganism growth	passed	passed

**Fig. 5:**
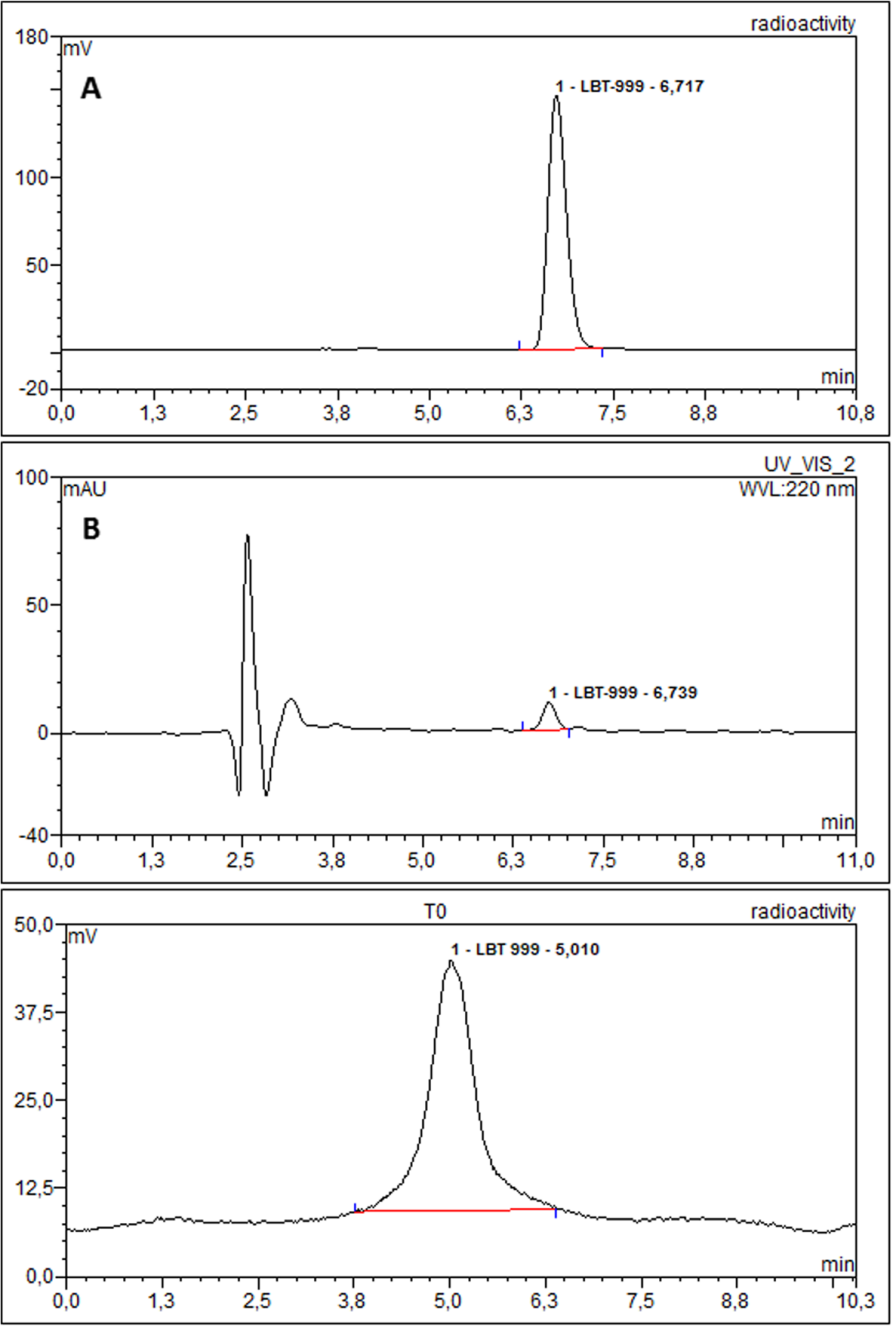
A/ and B/ Chromatograms (gamma and UV) obtained with a μBondapak™ column at the end of [^18^F]LBT999 production on the TRACERlab FXFN. C/ Radio-TLC chromatogram of [^18^F]LBT999 radiopharmaceutical at the end of production

**Fig. 6:**
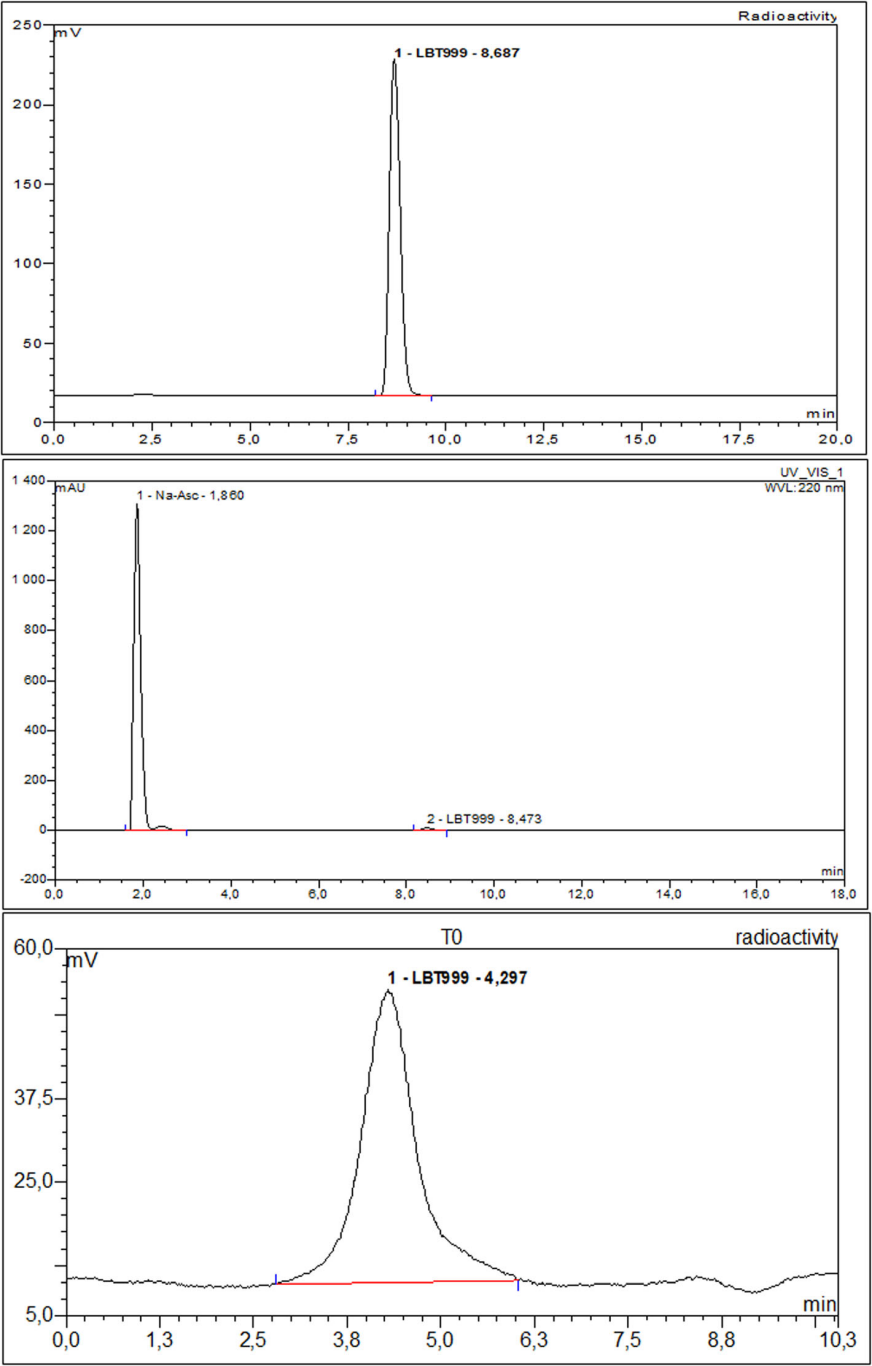
A/ and B/ Chromatograms (gamma and UV) obtained with a Bronwlee CHOICE C18 column at the end of [^18^F]LBT999 production on the AIO. C/ Radio-TLC chromatogram of [^18^F]LBT999 radiopharmaceutical at the end of production

The product was clear and free of particle matter, and the pH was compatible with human injection. The high chemical and radiochemical purities were confirmed by HPLC and TLC analysis (Figs. 5 and 6). The radiochemical purity of [^18^F]LBT999 was greater than 98% and no significant traces of free fluorine-18 or of the side product were observed by HPLC or TLC. The method allowed us to produce [^18^F]LBT999 with an average molar activity above 154 GBq/μmol and a radiochemical stability to at least 8 h (> 98%). Controls were also carried out 20 h after production without any loss of the specifications (data not shown). Bioburden tests were also performed both for TRACERLAB FXFN and AIO productions and met the required specifications.

## Conclusion

We report here the first robust and efficient automated radiosynthesis of [^18^F]LBT999 on two commercial platforms, TRACERlab FXFN and AIO for routine clinical use in GMP facilities. The same one-step radiosynthesis process was developed on both synthesizers and was validated for human use in clinical studies. The preparation of [^18^F]LBT999 was performed with good reproducibility, high radiochemical yield and high product quality. AIO proved to be more advantageous than TRACERlab FXFN as it can more easily comply with GMP and will be better adapted to industrial production. After optimizations on the AIO platform, we were able to significantly increase the radiochemical yield and to shorten the production time, thus making this the best method for a repeatable and robust production of [^18^F]LBT999. This automation of [^18^F]LBT999 can facilitate multicenter trials and widespread use of this radiopharmaceutical for DAT imaging by PET, and transfer onto other commercial modules using single-use cassettes may be considered.

## Data Availability

Please contact the corresponding author for data requests.

## Abbreviations

DAT: Dopamine Transporter
GMP: Good Manufacturing Practice
HPLC: High-Performance Liquid Chromatography
LBD: Lewy Bodies Dementia
Na-Asc: Sodium ascorbate
PET: Positron Emission Tomography
QC: Quality control
SPECT: Single Photon Emission Computed Tomography
TLC: Thin layer chromatography
